# Bidirectional associations of physical activity and cognitive function in midlife adults: a longitudinal analysis across 26 years follow-up

**DOI:** 10.1093/aje/kwaf144

**Published:** 2025-07-03

**Authors:** John J Mitchell, Mark Hamer, Sarah N James, Tom Norris, Barbara J Jefferis, S Goya Wannamethee, Joanna M Blodgett

**Affiliations:** Division of Surgery and Interventional Sciences, Faculty of Medical Sciences, Institute of Sport Exercise & Health, University College London, London, United Kingdom; Department of Primary Care and Population Health, Upper Third Floor UCL Medical School (Royal Free Campus), Rowland Hill Street, London NW3 2PF, United Kingdom; Division of Surgery and Interventional Sciences, Faculty of Medical Sciences, Institute of Sport Exercise & Health, University College London, London, United Kingdom; Biomedical Research Centre, University College London Hospitals NIHR, London, United Kingdom; MRC Unit for Lifelong Health and Ageing at UCL, University College London, London, United Kingdom; Division of Surgery and Interventional Sciences, Faculty of Medical Sciences, Institute of Sport Exercise & Health, University College London, London, United Kingdom; Department of Primary Care and Population Health, Upper Third Floor UCL Medical School (Royal Free Campus), Rowland Hill Street, London NW3 2PF, United Kingdom; Department of Primary Care and Population Health, Upper Third Floor UCL Medical School (Royal Free Campus), Rowland Hill Street, London NW3 2PF, United Kingdom; Division of Surgery and Interventional Sciences, Faculty of Medical Sciences, Institute of Sport Exercise & Health, University College London, London, United Kingdom; Biomedical Research Centre, University College London Hospitals NIHR, London, United Kingdom

**Keywords:** physical activity, dementia, cognitive decline, mild cognitive impairment, bidirectional, reverse causation

## Abstract

Prior studies linking physical activity (PA) and cognition typically assume a causal association between PA and subsequent cognition. Yet, there remains speculation regarding the direction of this association. We investigated bidirectional associations between PA and cognition. Participants of the Medical Research Council National Survey of Health and Development cohort, all born in 1946 reported their PA frequency, undertook processing speed and word recall memory tasks throughout midlife (ages 43 years(*y*), 53*y*, 63*y,* and 69*y*). There was evidence of bidirectional associations in initial structural equation models. To quantify this relationship, mixed-effects models were fitted with a lagged predictor and controlling for childhood cognition, socioeconomic and health factors, attrition, and mortality. Among 2888 participants (51% female), we report bidirectional associations between cognition and PA in midlife. A 1-standard deviation increase in verbal memory was associated with an increased probability of being in the mid-active category at the subsequent wave for females (relative risk ratio [RRR], 1.30; 95% confidence interval [CI], 1.15–1.46), while becoming active was associated with a minimally greater subsequent verbal memory *z* score (β = 0.08; 95% CI, 0.01–0.14). Bidirectional associations proved more robust for males. Results suggest that reciprocal associations exist between PA and cognition, yet stronger in the direction of cognition to PA.

## Introduction

Consensus is building as to the importance of adopting a lifelong healthy lifestyle for healthy cognitive aging.^1^^,^^2^ Mounting evidence has implicated physical activity (PA) as one such potentially modifiable lifestyle factor for reducing cognitive decline and risk of dementia in later life.^1^^,^^3^^,^^4^ There may be cumulative benefit from sustained PA across the life course for preserved cognitive health and slowing cognitive decline.^5^^,^^6^

Acute exercise is hypothesized to enhance cognitive function^7^^,^^8^ via several pathways, including increased brain-derived neurotrophic factor (BDNF) release, enhanced or preserved hippocampal neuroplasticity, and improved vascular function, which, in the longer term, may build cognitive reserve.^9^^-^^11^ There are also more acute cognitive benefits downstream of PA, from improved blood-glucose control and enhanced sleep.^11^ Finally, there may be wider cognitive benefits from PA of different modalities, which involve coordination, planning, and social components.^12^ However, reverse causation remains a major challenge when studying health conditions with long latency periods. Further, assuming a consistent, unidirectional relationship between PA and cognition across the life course may be overly simplistic and hence the direction of association between PA and cognition has remained contentious.^13^ For example, studies have also found an inverse relationship in later life,^13^^-^^18^ whereby more rapid cognitive decline may result in lower engagement in PA.^19^ Cognitive decline can lead to increased apathy, loss of motivation as well as the ability to plan effectively, leading to a gradual disengagement from activities.^14^^,^^19^^,^^20^ It is therefore plausible that a reciprocal relationship exists between PA and cognitive function in later life. The problem of reverse causality challenges the validity of claims regarding the protective role of physical activity (PA) (which is now promoted earlier in midlife) to counter age-related decline.^21^ The efforts and associated costs from interventions promoting PA may of limited effectiveness. It is, as yet unclear whether this bidirectional association exists earlier in life, when the rate of decline in cognitive resources is slower and clinical signs of impairment are not yet manifest. Equally, the presence of a strong association in the direction of PA to subsequent cognition would support the evidence favoring early promotion of PA to support long-term cognitive health.

Understanding the degree of reciprocity in the relationship between midlife PA and cognition is a crucial initial step toward determining the degree of reverse causality, which may partly underpin the positive associations reported in midlife and is fundamental to future intervention efforts. Previous studies investigating possible reciprocity in the relationship between cognition and PA have reported mixed findings, though do hint at the existence of some reverse causality, specifically in later life.^14^^,^^20^^,^^22^ One study identified no reciprocity whatsoever,^15^ while others observed a reciprocal relationship which was stronger in the direction of delayed recall or processing speed to subsequent levels of moderate PA.^14^^,^^22^ As outlined above, neither study has examined this association during midlife, and thus prior to the prodromal phase of dementia, nor accounting for childhood cognitive function, a major contributor to cognitive reserve.^23^ Further, few cited studies have applied statistical methods, such as cross-lagged approaches which formally assess both directions in tandem, despite their routine application in other fields.^24^^,^^25^

This study aims to use longitudinal data to explore the temporal order of the associations between PA and cognition over a 25-year period in midlife with the following aims: To assess the degree of reciprocal associations between PA and cognition across four points in midlife (ages 43 years [y]; 53y, 63y, and 69y) and (ii) whether associations are independent of known confounders?

## Methods

### Study design and sample

Participants were drawn from the Medical Research Council (MRC) National Survey of Health and Development (NSHD), an age-homogenous, nationally representative sample of 5362 individuals born within 1 week in 1946 across England, Scotland, and Wales.^26^ Study members have been regularly followed from birth and provided informed consent at each follow up. Ethical approval for the NSHD study was provided by Research Ethics Committees in England and Scotland.^27^ Participants completed regular health and lifestyle surveys which included self-reported PA and cognitive assessments and undertook anthropometric assessments at ages 43y, 53y, 63y, and 69y. Participants were eligible for these analyses if they had complete cognitive and PA data at ≥2 waves.

### Physical activity

Leisure time PA frequency was self-reported at ages 43y, 53y, 63y, and 69y by asking a similarly phrased question around: *“In the last 4 weeks, in your spare time, have you taken part in any sports or vigorous leisure activities or done any exercises, things like badminton, swimming, yoga, press-ups, dancing, football, mountain climbing or jogging?”*. Respondents answering “yes” were then asked, “*On how many occasions in the last month did you do these activities?*”. At age 43y response options were “less than once a month,” “less than once a week,” “once a week,” and “more than once a week.” At ages 53y, 63y, and 69y, participants instead reported the number of occasions per month (max 100). In line with previous studies in this cohort,^5^ responses were categorized as Inactive: “Never or less than monthly”; Moderately active: “1–4 times monthly”; Most active:“≥5 monthly”, groupings which have been shown to be associated with a number of health outcomes.^5^^,^^28^^-^^30^

### Cognition

At ages 43y, 53y, 63y, and 69y, cognition was assessed using tests of verbal memory and processing speed. Both tests have been widely used to assess cognition in large epidemiologic studies and are predictive of subsequent cognitive impairment.^31^^-^^34^

Verbal memory was tested using the word-learning task, a bespoke task designed for and routinely used in NSHD, but which aligns with similar verbal memory paradigms.^35^ This involves 15 words being shown to participants for two seconds each. Participants then recalled as many of these as possible by writing them down in any order. This was repeated across three trials and summed to produce a maximum score of 45. The word list was randomized between participants from two lists which were rotated between waves to diminish practice effects.^32^^,^^36^

Processing speed was assessed with a dual-letter search task in which participants search through a grid of 600 random letters, crossing out the target letters (Ps and Ws). The positions of target letters were different between waves.^37^ Processing speed was derived as the total number of letters screened within the 1-minute window.^33^

For interpretability, participant cognition scores were converted to *z* scores.

### Confounders

Confounders were selected a-priori to account for factors strongly associated with both PA and cognition^3^ and are outlined in detail in Appendix S1. Time-invariant confounders included *Educational Attainment* by age 26y^5^, *Parental Socioeconomic Position* measured using the Registrar General’s Social Classification^38^ and *Childhood Cognition*, measured at age 8 involving a battery of standardized tests.^5^^,^^39^ To account for the non-random dropout from mortality and attrition, additional adjustment was made for attrition, and mortality between ages 43y and 69y to account for possible survivorship bias.^40^ Ethnicity was not included in models, due to the vast majority of NSHD participants (>95%) being of white British ethnic background.^41^

Time-variant confounders included self-reported *smoking status*, *history of diabetes*, and history of *cardiovascular disease* (encompassing myocardial infarction, angina, and stroke). Each were collected at age 43y and updated at each subsequent wave.

### Statistical analyses

The presence of reciprocal paths between PA and cognition was first assessed using a structural equation modeling (Bivariate autoregressive cross-lagged panel [ARCLP] model) approach (see Figure S1). ARCLP models examine how each variable at one time point is predicted by its own prior values (autoregressive paths), as well as the prior values of other variables (cross-lagged paths) and their covariance at each age, in order to infer temporal precedence.^42^ Evidence of cross-lag paths provides evidence of probable underlying bidirectionality. Given prior evidence reporting sex differences in the association between cognitive function and PA engagement,^43^ “sex-grouped” models were fitted producing separate estimates. Modeling was performed in MPLUS.^44^ Further details of model-building and selection are outlined in the supplementary material (further details of model building and interpretation are outlined in Appendix S2, Figures S1 and S2, Table S1). To overcome the challenges of introducing covariates to ARCLP models, and the many imposed constraints and assumptions of these models, mixed-effects models were subsequently fitted to assess the magnitude of association in the presence of covariates.

Given observed bidirectional paths linking cognition and PA, mixed-effects models were used to quantify these associations. Mixed-effects models with individual-specific intercepts only, examined these same associations but between the independent variable at wave *w* and the dependent variables at wave *w* + 1 (ie, single time-point lag), with adjustment for baseline covariates.^14^ Separate models were fitted for each sex. Linear mixed-effects models were fitted for models where cognition was the dependent variable. Multinomial mixed-effects models were fitted where PA was the dependent variable and estimates reported as relative risk ratios (RRR). Non-linear time terms and interactions between the independent variable and time terms were tested. Adjustments were then made for baseline confounders (childhood cognition, SEP, and education) and midlife confounders: smoking status, history of CVD and diabetes.

Missing covariate data were imputed using *jomo* R package,^45^ a joint-modeling approach for imputing hierarchical data with a mixture of variable types.^46^ The imputation equation included all variables included in the mixed-effects models. The highest degree of missingness was observed in the age 69y wave history of CVD (20.7%) and diabetes (13.5%) and estimates were therefore pooled across 20 imputed data sets using Rubin’s rules^47^ (Table S2).

### Sensitivity analysis

In lagged mixed-effects models, we assessed an interaction between the exposure and a signifier of the final time lag. This approach helped account for possible differences which may be attributable to the short lag between age 63y and 69y.

## Results

The study sample consisted of 2888 participants (51% female (*n* = 1467) and 49% male (*n* = 1421)). Males had higher educational levels than females with 44.5% of males attaining A-levels or equivalent (attained at age 18) compared to just 29.4% of females. Additional participant characteristics are provided in Table 1. Differences in cognition scores over time by categorical PA levels are presented in Figures S3 and S4.

**Table 1:** Participant characteristics at age 43.

		Female		Male	
**Total**	N (%)	1467 (51%)		1421 (49%)	
**Sociodemographic factors**					
Parental social class	N (%)				
IV Partly Skilled/V-unskilled manual		342	(24.7%)	324	(23.9%)
III-Skilled non-manual or manual		677	(48.8%)	647	(47.8%)
I-Professional/II-Intermediate		368	(26.5%)	380	(28.3%)
**Childhood confounders & education**					
Age 8 standardized cognition score	Mean (SD)	0.09	(0.8)	0.06	(0.8)
Maximal educational attainment	N (%)				
None/below O-level/GCSE (less than age 16)		482	(34.7%)	465	(34.4%)
O-level/GCSE or vocational qualification		498	(35.9%)	285	(21.1%)
A-level or equivalent (Up to age 18)/ degree or higher degree		409	(29.4%)	600	(44.5%)
**Health & lifestyle factors**					
Smoker status	N (%)				
Never		476	(33.5%)	325	(26.1%)
Ex-smoker		668	(40.0%)	611	(45.0%)
Current smoker		376	(26.5%)	391	(28.9%)
History of diabetes	N, (%)	11	(0.7)	13	(0.9)
History of CVD	N, (%)	44	(3.2)	43	(3.2)
Deceased by age 69y	N (%)	110	7.5	149	10.5
Attrition by age 69y	N (%)	114	7.7	136	9.5

Reported percentages are prior to imputation and therefore totals for each covariate vary depending on missingness.

### Autoregressive cross-lagged panel models

**Figure 1:**
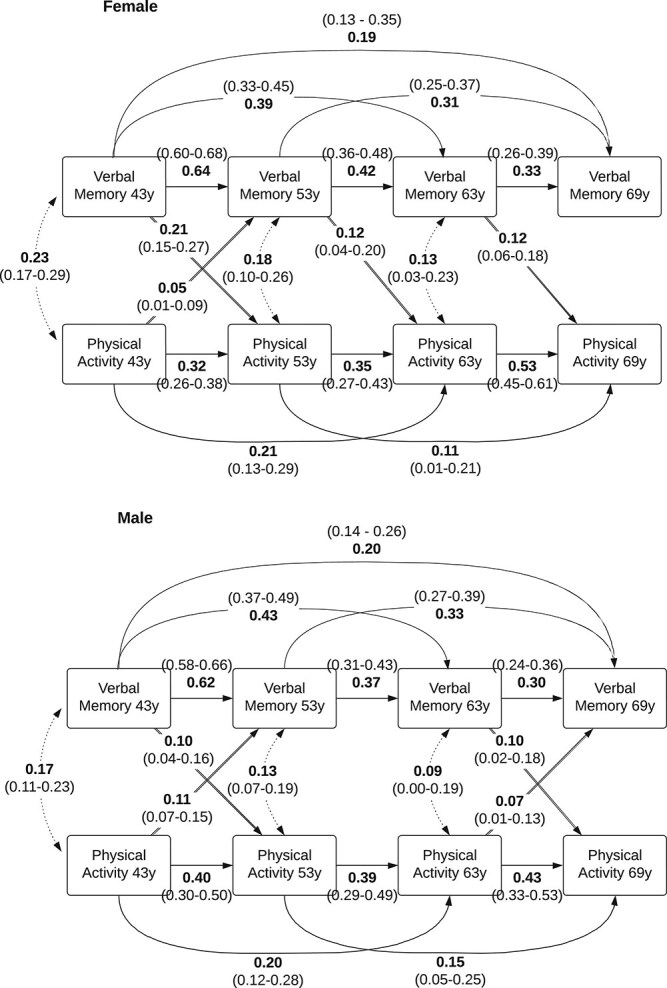
Standardized estimates of the associations between verbal memory and physical activity (PA) across ages 43y, 53y, 63y, and 69y using bivariate autoregressive cross-lagged panel models. Estimates for PA on subsequent cognition are for a 1SD increase on the continuous latent factor PA^*^. The corresponding SD thresholds for transition between categories on the observed PA variable are presented in Supplementary Table S5. Only significant paths including covariances are displayed.

The model-building process including final model fit indices are summarized in Table S1. Cross-lagged panel models revealed evidence of bidirectional paths linking PA and cognition. Across all models, the presence of paths between cognition and subsequent PA proved more consistent than paths linking PA with subsequent cognition, and particularly when comparing the first two time points (age 43y on 53y) with the final time points (age 63y on 69y) (Figure 1; Tables S3 and S4; transition thresholds for the PA variable are presented in Table S5). However, minimal evidence of lasting associations between PA and processing speed was observed, in either direction, for both sexes, in particular females using the cross-lagged panel approach (Figure S5).

### Mixed-effects models

Given the initial evidence of bidirectional paths linking PA and cognition within midlife participants, mixed-effects models were used to re-examine and quantify the magnitude of the observed relationships in the presence of possible confounding factors. These models included the independent variable lagged by one time point to ensure longitudinal exploration.

### Association of verbal memory and subsequent physical activity

**Figure 2:**
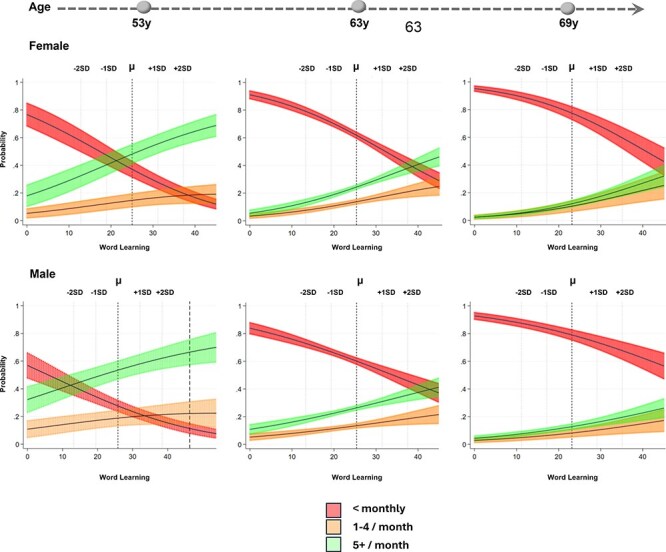
Predicted probability (predictive margins) of physical activity category at follow-up wave (*w*) by lagged verbal memory (word learning task *z* score) (at wave *w-1*) from multinomial mixed-effects models with 95% confidence intervals.

In unadjusted models in females, verbal memory was positively associated with subsequent PA. In comparison to individuals who exercise less than once a month, a 1-standard deviation (SD) was associated with a greater probability of being moderately active at the subsequent wave (RRR, 1.63; 95% CI, 1.47–1.82; most active: RRR, 1.86; 95% CI, 1.66–2.09; Figure 2; Table S6). This persisted when adjusted for childhood factors, education, adult health, attrition and mortality (moderately active: RRR, 1.30; 95% CI, 1.15–1.46); most active: RRR, 1.48; 95% CI, 1.31–1.68).

**Figure 3:**
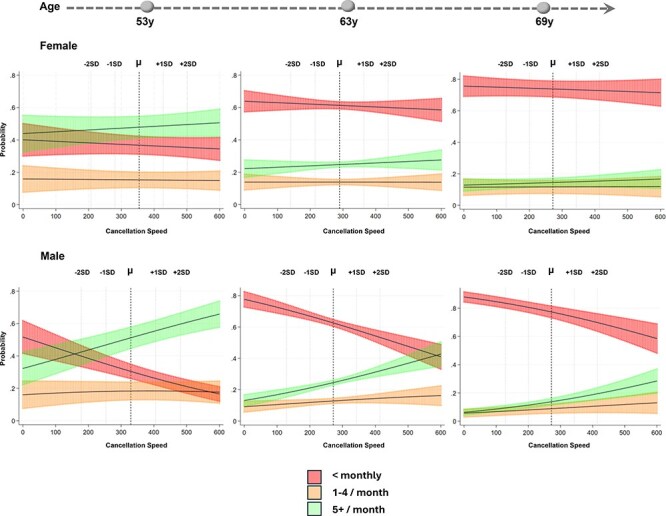
Predicted probability (predictive margins) of physical activity category at follow-up wave (*w*) by lagged processing speed (cancellation speed task *z* score) (at wave *w-1*) from multinomial mixed-effects models with 95% confidence intervals in male participants.

In male participants, this relationship was less pronounced, though a 1-SD increase in the previous verbal memory score was linked to greater subsequent PA (moderately active: RRR, 1.48; 95% CI, 1.32–1.67; most active: RRR, 1.63; 95% CI, 1.44–1.84); Figure 3; Table S6) and persisted after full adjustment (moderately active: RRR, 1.24; 95% CI, 1.10–1.41; most active: RRR, 1.30; 95% CI, 1.15–1.48).

### Association of physical activity and subsequent verbal memory

**Figure 4:**
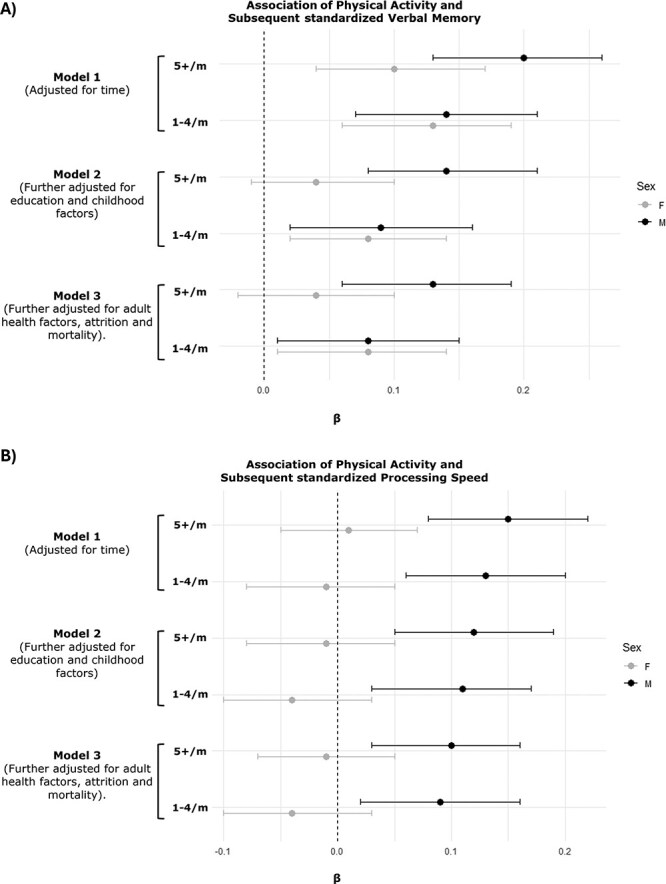
Associations between lagged physical activity and subsequent verbal memory *z* scores (A) and processing speed *z* scores (B). Model 1 (M1) adjusts for time (centered at age 53), and time squared (reference: none/<monthly). Model 2 (M2) further adjusts for education, childhood cognition and socioeconomic position. Model 3 (M3) further adjusts for adult health and lifestyle including cardiovascular disease, diabetes and smoking status, attrition and mortality.

For the inverse association, in female participants, relative to inactive individuals, moderate engagement in PA was weakly associated with greater recall in unadjusted models (moderately active: β, 0.13; 95% CI, 0.06–0.19); most active: β, 0.10; 95% CI, 0.04–0.17) Figure 4, Table S7). However, this only persisted for the moderately active group after full adjustment (β,0.08; 95% CI, 0.01–0.14).

Similar associations were observed in males in unadjusted (moderately active: β, 0.14; 95% CI, 0.07–0.21; most active: β, 0.20; 95% CI, 0.13–0.26) and adjusted (moderately active: β, 0.08; 95% CI, 0.01–0.15; most active: β, 0.13; 95% CI, 0.06–0.19) models.

### Association of processing speed with subsequent physical activity

In alignment with initial ARCLP models, there was limited evidence of associations between processing speed and PA in either direction for females. For males, a 1-SD processing speed was weakly associated with higher PA engagement at the next wave (moderately active: RRR, 1.33; 95% CI, 1.16–1.54); most active: RRR, 1.76; 95% CI, 1.51–2.04). This association persisted in fully adjusted models (moderately active: RRR, 1.19; 95% CI, 1.02–1.37); most active: RRR, 1.50; 95% CI, 1.30–1.74).

### Association of physical activity and subsequent processing speed

In the inverse direction, greater prior PA levels were positively associated with processing speed (moderately active: β, 0.13; 95% CI, 0.06–0.20; most active: β, 0.15; 95% CI, 0.08–0.22). These associations were partially attenuated in fully adjusted models (moderately active: β, 0.09; 95% CI, 0.02–0.16; most active: β, 0.10; 95% CI, 0.03-0.16).

### Sensitivity analysis

Despite no interactions with the linear time variable, a positive interaction was observed between a binary marker of the age 69 years wave and lagged verbal memory (*P* < 0.05; Tables S8 and S9) revealing a subtly stronger association with PA in males at this time point due to the shortened time lag.

## Discussion

This large-scale longitudinal study spanning 26 years aimed to examine the directionality of the associations between PA and cognitive function across midlife. We report a weak bidirectional relationship between PA and verbal memory that is stronger in the direction of cognition on subsequent PA. A 1-SD increase in verbal memory was linked with a 10% to 46% greater probability of being in a more active category in the subsequent wave for females, and 15% to 48% greater probability for males. Meanwhile relative to inactive individuals, engaging in PA at least monthly corresponded to an approximately 0.08-SD greater recall at the next wave in both males and females. These results remained robust to adjustment for confounding factors including major proxies of cognitive reserve such as education levels, childhood IQ and social class, as well as health and lifestyle factors including cardiovascular disease and smoking status, and markers of attrition or mortality within the study period. These results reveal an association between PA and cognition as far back as middle age, though this relationship appears to differ by cognitive domain, with a stronger reverse relationship, particularly verbal memory in females, and a more reciprocal relationship between PA and processing speed in males.

This study addresses two major issues in prior studies by accounting for the inverse association and adjusting for early life cognition.^4^^,^^48^ The few studies directly examining the underlying reciprocity of cognitive function and PA relationship to date report differing conclusions,^14^^,^^15^^,^^22^ revealing either no reciprocity,^15^ or a weak but reciprocal relationship.^14^^,^^22^ This study aligns with the latter studies but using two modeling approaches which help to account for the limitations of the other (modeling both relationships in tandem, and also accounting for individual-level differences and covariates). This study improves reliability by using the same measures of PA (intensity/frequency or context) and cognitive function (different functions) across waves, and over a longer time span. Our study extends prior studies by almost a further decade into midlife, in an age homogenous cohort, corroborating a similar, nuanced bidirectional relationship principally for verbal memory.

We observed positive associations between PA and subsequent cognition, but with substantive sex differences. Despite bidirectional associations for verbal memory, there was not clear evidence across both models comparing models of the presence of bidirectional associations between PA and processing speed, particularly for females. Mixed-effects models did however identify weak associations in males, in alignment with prior studies in this cohort.^5^ Importantly, the use of ARCLP models relies on null hypothesis significance testing in the identification of paths, which may result in the constraint of paths which were tending toward significance at the pre-defined threshold. The subtle sex-differential findings for the associations between PA and subsequent cognition may be crucial for future studies and warrant further investigation to better understand biological or psychosocial reasons that may underlie these differences. Studies to date have revealed sex differences in a number of implicated pathways linking PA of a moderate-to-vigorous intensity and cognition including the BDNF pathway and a number of circulating hormones with downstream neural effects.^43^ However, there is also emerging evidence which implicates PA context as a likely moderator of any association, specifically for processing speed.^12^ Benefits to processing speed appear more strongly related to specific modes of PA, such as skill acquisition in sports participation,^12^^,^^49^ contexts which may be more routinely practiced by males throughout life.^12^^,^^50^ Few studies to date have accounted for the importance of such a distinction in this relationship^12^^,^^51^ and future studies may apply similar methodologies while accounting for the differing contexts and intensities of PA performed between sexes.

Associations in the inverse direction were also observed (ie, earlier cognition being associated with subsequent PA levels) in both sexes a finding also observed in other stages of life,^52^ but which is often overlooked or unaccounted for in studies in older adults. This relationship, specifically in older adults, is often explained by theories describing the loss of motivation, inhibition and organizational abilities which accompanies cognitive decline.^14^ Inhibition appear essential for counteracting the tendency toward effort minimisation.^19^ Further, as cognitive decline becomes more clinically manifest, the capacity of the individual to maintain effortful activities including organization, planning and socializing may also diminish.^19^^,^^20^ Finally, individuals with greater cognitive capability are also thought to exhibit a better general health literacy which supports their awareness of the needs to exercise,^53^ though adjustments for education in this study did not entirely curtail observed relationships.

Our study broadly supports current trials of interventions of PA in midlife which aim to enhance cognitive ability directly as well as promote PA^54^^,^^55^ given the observed reciprocity of the association. However, we observed only small associations which differed between sexes and an overall larger inverse association. The implications of this finding are also critical for re-interpreting the extant literature examining this pathway. Prior studies using unidirectional modeling approaches may overestimate the causal impact of PA on cognitive function, if underlying cognitive capacity is strongly influencing engagement in activity. A more tempered interpretation may therefore by warranted. This finding has practical implications for future research. First, this bidirectional association must be accounted for in future studies examining the PA–cognition relationship. Second, we speculate that the relative benefits from such interventions, and the effective components of interventions may be small at the level of the individual, change across time, and differ between sexes. Therefore, we hypothesize that combined interventions which focus on preserving cognitive function in other ways may slow the gradual age-related onset of physical inactivity, which also serves to maintain cognition.

### Strengths and limitations

Our study utilizes the largest, continuously running, nationally representative birth cohort to examine the temporal ordering of the PA-cognition association across a period spanning over 25 years. The methodology combined mixed-effects models with a further approach which directly examines the reverse association directly rather than assuming the causal direction, the biggest limitation of preceding studies. Finally, this study explores this complex issue within midlife, starting almost a decade earlier than similar studies which helps to mitigate the risks of preexisting pathology. We acknowledge a number of limitations. First, the excluded sample differed on several covariates from our sample (Table S10), which may lead to selection bias. Inclusion of these individuals who were proportionally more male, had lower educational attainment, and generally worse midlife health may have led to weaker estimates. Second, measures of PA within NSHD are necessarily collapsed to trichotomous measures of frequency for harmonization across time, reducing the granularity of our findings. Further, these PA frequency measures do not account for the contexts or intensity of PA which are likely to be important in shaping this relationship. Further, while reliant on these repeated PA questions and cognition paradigms, used by the NSHD study over recent decades, there is inherent, widely-reported issues with self-reported PA measures, including among older adults.^56^ Misclassification within the PA variable could invariably bias effect estimates in either direction. Similarly, while rotation of word lists was performed every other follow-up, it is not possible to rule out possible learning effects.^36^ While the cognitive test paradigms used in this study have been widely used, individual performance on the day of testing may still be confounded by acute stress or other sources of residual confounding. Other sources of bias also exist. While the longitudinal modeling strategies employed attempt to account for possible underlying reciprocity, recent studies have discussed how these approaches may inadvertently introduce collider bias affecting the cross-lagged estimates, and could not be mitigated.^57^^,^^58^ Finally, future studies may opt to adopt similar, innovative reciprocal modeling approaches, such as causal discovery methods,^59^^,^^60^ but with more granular PA measures, and a wider variety of cognition paradigms to corroborate our findings in alternate samples.

## Conclusion

Given the emerging role of midlife PA for bolstering cognition and thereby building resilience to cognitive decline, this study sought to examine the temporal dynamics of this association. We report the presence of bidirectional associations between PA and cognition in midlife though to a greater extent in males, and which is independent of measured confounding factors. However, this relationship varied by cognitive domain, sex and timing in the life course. Despite modest associations, our findings support the evidence base favoring a role of PA in supporting certain facets of cognition from as early as midlife, though critically, our findings place an onus on future studies to account for reverse causation, and to test sex-specific differences when examining risk factors for cognitive decline.

## Supplementary Material

Web_Material_kwaf144

## Data Availability

Data are available on reasonable request. Data used in this publication are available to bona fide researchers on request to the NSHD Data Sharing Committee via a standard application procedure. Further details can be found at http://www.nshd.mrc.ac.uk/data. doi:10.5522/NSHD/Q102; 10.5522/NSHD/Q103.
