# Supplementary material for: Bidirectional associations of physical activity and cognitive function in midlife adults: a longitudinal analysis across 26 years follow-up
Source: Am J Epidemiol. 2025 Jul 3;194(9):2514–23. doi: 10.1093/aje/kwaf144 (PMC12799601; doi:10.1093/aje/kwaf144)
Supplement: Web_Material_kwaf144 [file web_material_kwaf144.docx]

**Supplementary Material:** Bidirectional associations of Physical Activity and Cognitive Function in Midlife Adults: A Cross-Lagged Panel Analysis.

Authors: Dr John J Mitchell, Professor Mark Hamer, Dr Sarah N James, Dr Tom Norris, Dr Barbara J Jefferis, Professor S Goya Wannamethee, Dr Joanna M Blodgett.

Index:

**Appendix S1 -** Covariate Coding Details

**Supplementary Figure S1 -** ARCLP model structure

**Appendix S2 -** Autoregressive Cross-lagged Panel Model-building approach

**Supplementary Figure S2 -** Model Building Approach

**Supplementary Table S1 -** Model Building Procedure, Fit Indices and Chi Square Tests of nested, constrained models

**Supplementary Table S2 -** Missingness by Wave

**Supplementary Figure S3 -** Verbal memory and Processing speed scores

**Supplementary Figure S4** - Change in cognitive scores over time

**Supplementary Table S3** - Bivariate CLPM of PA and Verbal memory (VM)

**Supplementary Figure S5 -** Final, constrained Autoregressive cross-lagged panel models (Processing Speed)

**Supplementary Table S4 -** Bivariate CLPM of PA and Processing Speed (PS)

**Supplementary Table S5** - Standardised Transition Thresholds of PA*latent Factor

**Supplementary Table S6 -** Associations between physical activity scores and lagged (prior) cognition

**Supplementary Table S7** - Associations between lagged (prior) physical activity and cognitive domain scores

**Supplementary Table S8** - Interactions of an age-69 indicator variable in random effects models

**Supplementary Table S9 -** Interactions of an age-69 indicator variable in random effects models

**Supplementary Table S10 -** Participant Characteristics at Baseline (age 43)

**Appendices S1. Covariate Coding Details**

*Time-invariant Confounders*

*Sex at birth* was reported at baseline and categorised as binary (0:Male, 1:Female). *Educational Attainment* was the highest level of education attained by age 26y and operationalised similar to previous studies as 1: none/below ordinary secondary qualifications (below age 16y) 2: vocational or ordinary secondary qualifications (up to age 16y), 3: advanced secondary qualifications (up to age 18y), degree or higher degree^1^. *Parental* *Socioeconomic Position* was measured using the Registrar General's Social Classification was used to categorise parental occupational class and coded as I-Professional/ II-Intermediate, III-Skilled non-manual or manual, IV-Partly skilled/ V-Unskilled manual^2^. *Childhood Cognition*, measured at age 8 involved tests of reading comprehension (sentence completion), reading ability (word-reading and pronunciation), vocabulary (understanding the meaning of 50 words) and non-verbal reasoning (picture intelligence task). Participant scores were standardised across all participants who took the four tests to produce a childhood cognition summary score^1, 3^.

Time-variant Covariates

Self-reported smoking status was reported by questionnaire at ages 43y, 53y, 63y and 69y and scored as ‘never’, ‘ex’ or ‘current’ smoker. Self-reported history of diabetes and cardiovascular disease (encompassing myocardial infarction, angina and stroke) were each scored as (yes/no). Each were collected at age 43 and updated at each subsequent follow-up.

**Supplementary Figure S1. – ARCLP model structure**


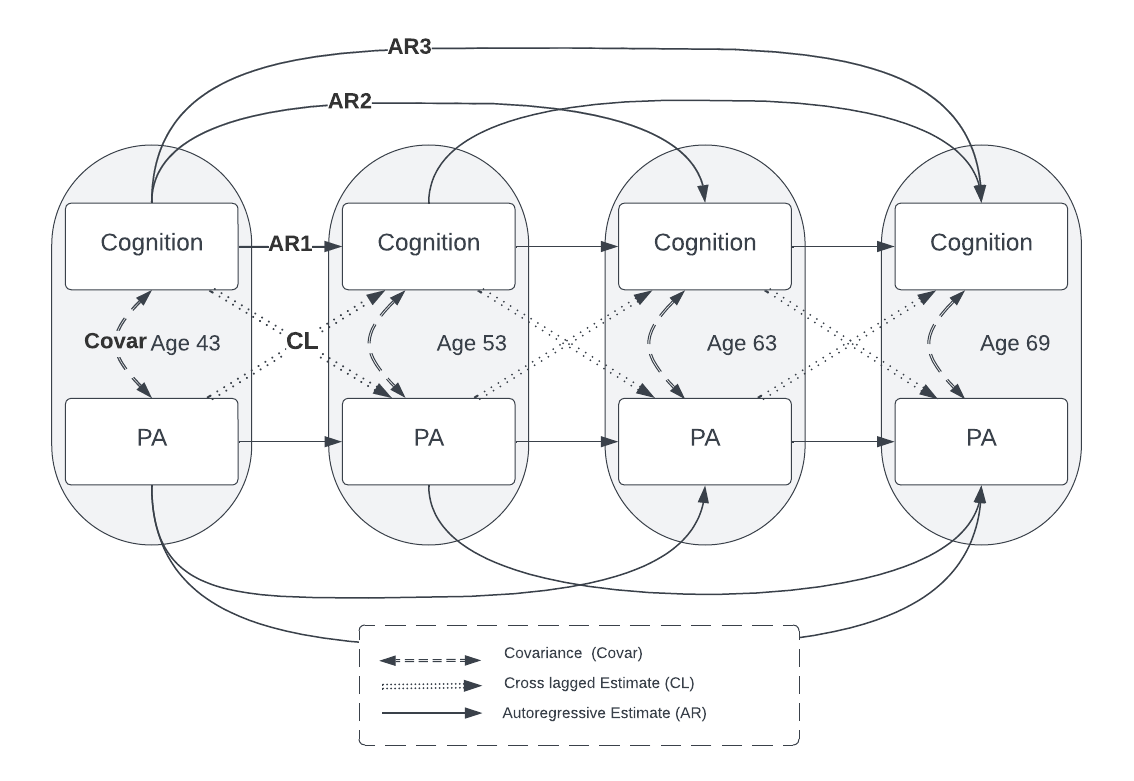


**Supplementary Figure S1. Autoregressive cross-lagged panel (ARCLP) model structure** to assess the relationship between baseline (wave 1) and follow-up measures (wave 2) of cognition and physical activity (PA). **Autoregressive paths (solid arrows):** Assess the stability of cognition scores and PA levels over time **Cross-lagged paths (dashed arrows):** Indicate the influence of wave 1 cognition on wave 2 PA and vice versa, while controlling for their respective baseline levels. **Covariance** (**dashed double-sided arrows):** Measure variance of the baseline measures and covariance between the residuals of the follow-up measures.

**Appendices S2: Autoregressive Cross-lagged Panel Model-building approach**

Autoregressive Cross-Lagged Panel (ARCLP) models analyse dynamic interactions and temporal relationships between variables. At the outset, the variance (Var) of each variable is assessed. There are three key pathways in each model: autoregressive, cross-lagged and covariance paths. Autoregressive paths indicate the stability of a variable, demonstrating how a variable at one time point predicts itself at subsequent points, controlling for its prior values. Covariance paths indicate how the two variables co-vary at the same time point. Cross-lagged paths examine directional influences, revealing how one variable at wave (*w*) can influence the other at a later time *(w+1).* By controlling for prior values of the variables, ARCLP models can indicate temporal precedence in the association between the two variables over time.

The modelling procedure consisted of two steps: (i) building a parsimonious base model based on fit indices, and (ii) testing imposed equality constraints using a series of nested models compared with the MPLUS DIFFTEST likelihood ratio test (LRT). Fit indices (reference values outlined below) used for model-building included root mean square error of approximation (RMSEA), Tucker-Lewis Index (TLI), Comparative fit index (CFI), Standardized Root Mean Square Residual (SRMR). The DIFFTEST LRT necessarily adopts null hypothesis significance testing, and compares the nested model m to the previous model with any retained constraints (m-1). Multi-group models were fit, grouping individuals by sex to produce separate estimates. Autoregressive cross-lagged panel models were fitted using the weighted least squares, mean and variance adjusted (WLSMV)^4-6^ estimator with probit-link function. These estimation procedures are used to account for the ordinal nature of the PA variable. When applied to the continuous cognitive outcomes, WLSMV estimates how a 1-unit difference in this variable is related to change in the other variables in the model, irrespective of their distribution and yields both unstandardized and standardized results. For ordinal PA however, the WLSMV estimator uses the probit link function to estimate a continuous latent factor (*y** i.e. ‘*underlying PA behaviour’*) based on the probability of being in each category of the observed ordinal PA variable (*y*) and produces thresholds/cut points on continuous factor *y** which map to the levels of the ordinal variables. A low value of  *y** (below the first of two cut-points) would indicate a probability of belonging to the first category of ordinal PA variable *y*. This continuous y* latent PA factor can be regressed with the cognitive outcomes and standardised to produce both standardised and unstandardised results.

| Fit Index | [good fit] Range |
| --- | --- |
| RMSEA | [0.00-0.10] 0-1 |
| CFI | [≥0.95] 0-1 |
| TLI | [≥0.90] 0-1 |
| SRMR | [≤0.05] 0-1 |

**Model assumptions**

Interval equality assumes that cross-lagged effects remain stable across all measurement periods, yet the relationship between each variable may evolve over time due to aging-related processes. This assumption was therefore necessarily relaxed when imposing/testing constrained models^7^.

While some feedback within intervals is inevitable due to the acute effects of PA on cognition, a suitable time lag was utilised (approximately 10y) allowed the focus of models to capture the long-term changes in cognition, and resultant change in PA *habits*.

The final model assumes linearity, which was imposed for brevity, and may not capture non-linear effects.

Finally, the presented models assume individuals share the same autoregressive and cross-lagged dynamics, potentially -though necessarily- simplifying this complex relationship. Convergence issues hindered the use of more flexible random-intercept approach. This approach is therefore bolstered by their combination with mixed-models.

**Interpretation**

For interpretation, cognitive outcomes were standardised to produce z-scores. In ACLP models, unstandardised coefficients are therefore interpreted as ‘the change in cognitive z-score (SDs) associated with a 1-category change in PA. However, in the opposite direction this is interpreted as ‘the change in continuous latent PA factor y* in relation to a 1-unit (1 standard-deviation) change in the cognitive score’. Standardised coefficients give this same output but both in terms of standard deviations. Thresholds indicate the cut-point at which an individual with this value of y* is most likely to move to the next category of observed PA variable y, allowing estimation of the necessary change in cognitive z-score to move an individual between categories of the observed ordinal PA variable y. The thresholds in STDYX are in standard deviations away from the mean of the continuous latent variable.

**Temporal Dynamics**

Equality constraints applied to significant bidirectional cross-lags were then tested to test for evidence of a prevailing direction of association. For example, the path from PA43 to Cog53 was constrained to be equal to the path from Cog43—>PA53. If model fit did not change/improve, this would suggest that the pathways did not differ. However if constraining this pathways resulted in worse fit, this would indicate a stronger association in one direction based on the size of the coefficients

**Final Models**

Third order-autoregressive structure with constrained first-order autoregressive PA pathways proved the most parsimonious base model for both word-learning task scores and Processing Speed, with constraints imposed on all significant paths in males improving model fit. Each model is summarised below. The model-building process including final model fit indices are summarized in Supplementary Table S1. The optimal models for both cognitive tests involved inclusion of a third-order autoregressive structure whereby each variable was associated with its own subsequent values at wave w+1, w+2 and w+3. All reported estimates are standardized and presented in figures 3 and 4.

**Supplementary Figure S2 - Model Building Approach.**


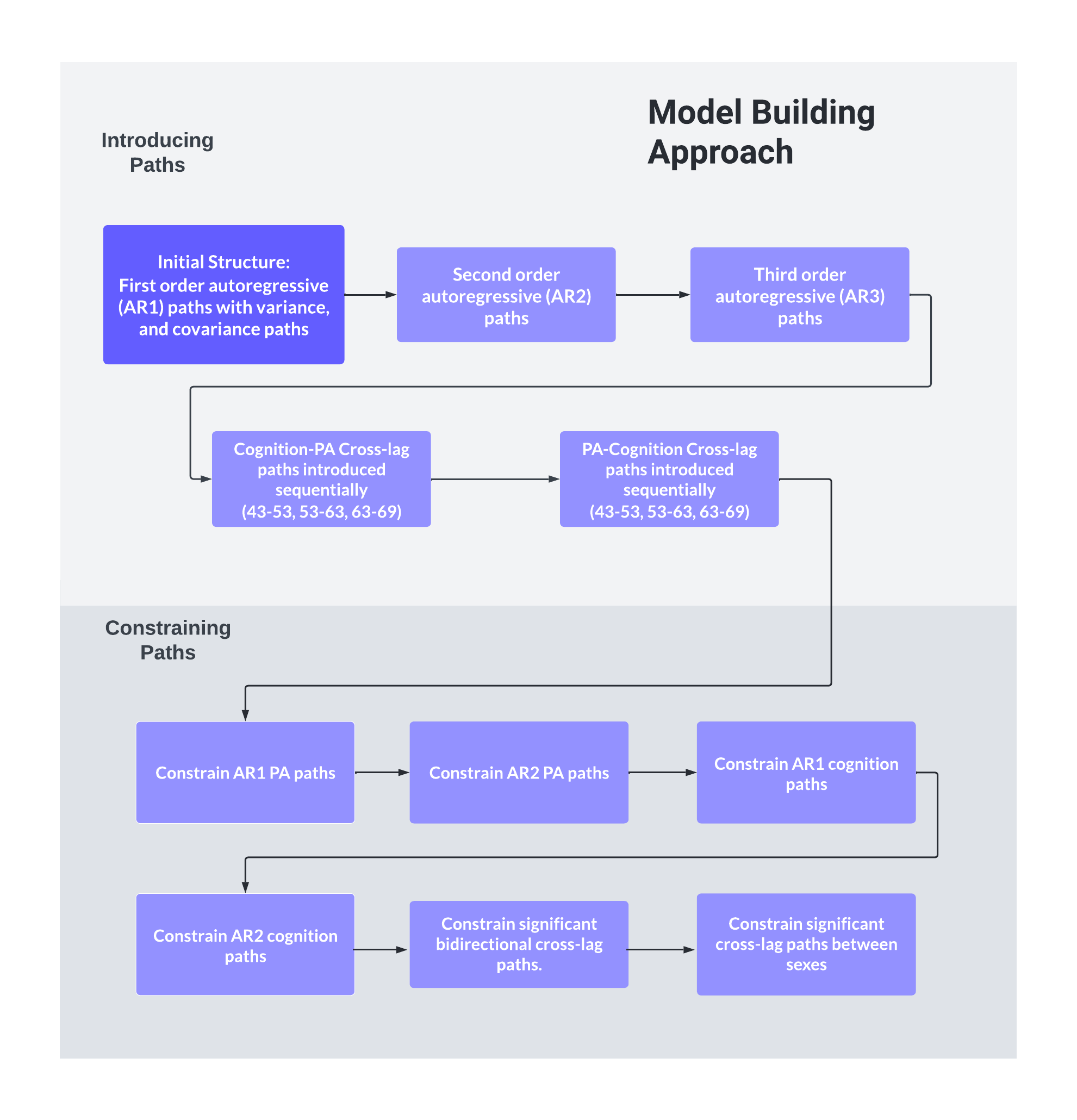


| **Supplementary Table S1. Model Building Procedure, Fit Indices and Chi Square Tests of nested, constrained models.** | | | | | | | | | |
| --- | --- | --- | --- | --- | --- | --- | --- | --- | --- |
| **Outcome** | **Models / Constraint** | **χ2** | **(df)** | **RMSEA** | **CFI** | **TLI** | **SRMR** | **Retained/Rejected** | **LRT p-value** |
| Word-Learning | **Introduction of Paths (Base Model)** |  |  |  |  |  |  |  |  |
|  | AR1, Variance, Covariance No Cross-lags | 452.622 | 36 | 0.09 | 0.897 | 0.84 | 0.088 | Retained | - |
|  | AR2 No Cross-lags | 195.566 | 28 | 0.064 | 0.959 | 0.917 | 0.076 | Retained | - |
|  | AR3 No Cross-lags | 167.794 | 24 | 0.064 | 0.964 | 0.917 | 0.077 | Retained | - |
|  | AR3 VERBAL MEMORY43-PA53 Cross-lags | 98.65 | 22 | 0.049 | 0.981 | 0.952 | 0.031 | Retained | - |
|  | AR3 VERBAL MEMORY53-PA63 Cross-lags | 69.493 | 20 | 0.041 | 0.988 | 0.966 | 0.023 | Retained | - |
|  | AR3 VERBAL MEMORY63-PA69 Cross-lags | 38.93 | 18 | 0.028 | 0.995 | 0.984 | 0.015 | Retained | - |
|  | AR3 PA43-VERBAL MEMORY53 Cross-lags | 21.82 | 16 | 0.016 | 0.999 | 0.995 | 0.012 | Retained | - |
|  | AR3 PA53-VERBAL MEMORY63 Cross-lags | 22.861 | 14 | 0.021 | 0.998 | 0.991 | 0.012 | Retained | - |
|  | AR3 PA63-VERBAL MEMORY69 Cross-lags | 18.157 | 12 | 0.019 | 0.998 | 0.993 | 0.01 | Retained | - |
|  | **Constraint of Autoregressive Paths** |  |  |  |  |  |  |  |  |
|  | Constrained AR1 PA | 32.634 | 16 | 0.027 | 0.996 | 0.986 | 0.014 | Rejected | 0.008 |
|  | Constrained AR2 PA | 28.274 | 14 | 0.027 | 0.996 | 0.986 | 0.014 | Rejected | 0.018 |
|  | Constrained AR1 VERBAL MEMORY | 104.708 | 16 | 0.062 | 0.978 | 0.923 | 0.026 | Rejected | <0.01 |
|  | Constrained AR2 VERBAL MEMORY | 22.631 | 14 | 0.021 | 0.988 | 0.991 | 0.011 | Rejected | 0.03 |
|  | **Constraint of Significant Cross-Lags** |  |  |  |  |  |  |  |  |
|  | PA43-53; VERBAL MEMORY43-53 (M only) | 17.222 | 13 | 0.015 | 0.999 | 0.996 | 0.01 | Retained | **0.531** |
|  | PA43-53; VERBAL MEMORY43-53 (F only) | 41.125 | 13 | 0.039 | 0.993 | 0.97 | 0.02 | Rejected | <0.01 |
|  | PA63-69; VERBAL MEMORY63-69 (M) | 21.997 | 14 | 0.02 | 0.998 | 0.992 | 0.012 | Retained | **0.08** |
|  | **Constraint of Significant Bidirectional Cross-Lags between Sexes** | | |  |  |  |  |  |  |
|  | PA43-53; VERBAL MEMORY43-53 (M+F equal) | 39.727 | 15 | 0.034 | 0.994 | 0.977 | 0.02 | Rejected | 0.002 |
|  |  |  |  |  |  |  |  |  |  |
|  | **Models / Constraint** | **χ2** | **(df)** | **RMSEA** | **CFI** | **TLI** | **SRMR** | **Retained/Rejected** | **LRT p-value** |
|  | **Introduction of Paths (Base Model)** |  |  |  |  |  |  |  |  |
| Processing Speed | AR1, Variance, Covariance No Cross-lags | 341.336 | 36 | 0.077 | 0.922 | 0.879 | 0.061 | Retained | - |
|  | AR2 No Cross-lags | 63.8 | 28 | 0.03 | 0.991 | 0.982 | 0.026 | Retained | - |
|  | AR3 No Cross-lags | 33.47 | 24 | 0.017 | 0.998 | 0.994 | 0.02 | Retained | - |
|  | AR3 Speed43-PA53 Cross-lags | 29.569 | 22 | 0.015 | 0.998 | 0.995 | 0.017 | Retained | - |
|  | AR3 Speed53-PA63 Cross-lags | 27.813 | 20 | 0.015 | 0.997 | 0.994 | 0.015 | Retained | - |
|  | AR3 Speed63-PA69 Cross-lags | 25.498 | 18 | 0.017 | 0.998 | 0.994 | 0.013 | Retained | - |
|  | AR2 PA43-Speed53 Cross-lags | 17.329 | 16 | 0.008 | 0.999 | 0.999 | 0.011 | Retained | - |
|  | AR2 PA53-Speed63 Cross-lags | 16.605 | 14 | 0.011 | 0.999 | 0.997 | 0.01 | Retained | - |
|  | AR3 PA63-Speed69 Cross-lags | 18.687 | 12 | 0.02 | 0.998 | 0.992 | 0.01 | Retained | - |
|  | **Constraint of Autoregressive Paths** |  |  |  |  |  |  |  |  |
|  | Constrained AR1 PA | 30.564 | 16 | 0.0153 | 0.996 | 0.987 | 0.015 | Rejected | <0.01 |
|  | Constrained AR2 PA | 26.144 | 14 | 0.025 | 0.997 | 0.988 | 0.015 | Rejected | 0.03 |
|  | Constrained AR1 Speed | 57.21 | 16 | 0.042 | 0.99 | 0.963 | 0.018 | Rejected | <0.01 |
|  | Constrained AR2 Speed | 33.887 | 14 | 0.031 | 0.995 | 0.98 | 0.014 | Rejected | <0.01 |
|  | **Constraint of Significant Cross-Lags** |  |  |  |  |  |  |  |  |
|  | Speed53; PA43-53 M | 17.646 | 13 | 0.016 | 0.999 | 0.995 | 0.011 | Retained | **0.681** |
|  | **Constraint of Significant Bidirectional Cross-Lags between Sexes** | | |  |  |  |  |  |  |
|  | - | - | - | - | - | - | - | - | - |
| Use of Likelihood ratio (MPLUS DIFFTEST) tests (reliant on testing significance) is a standard means of comparing nested model (*m*) to previously retained model (*m-*1). When imposing constrains, a significant DIFFTEST indicates loss of model (*m*) compared to the nested, constrained model (*m-*1). Abbreviations: X2: Chi Square; df: degrees of freedom; RMSEA: Root mean square error of approximation; CFI: Comparative Fit Index; TLI: Tucker-Lewis Index; SRMR: Standardized Room Mean Square Residual; LRT: Likelihood Ratio Test | | | | | | | | | |

| **Supplementary Table S2. Missingness by Wave** | **Age 43** | **Age 53** | **Age 63** | **Age 69** |
| --- | --- | --- | --- | --- |
| **Covariate** |  |  |  |  |
| Parental Social Class | 4.8% | 5.2% | 5.1% | 5.4% |
| Age 5 Cognition (Standardised) | 10.8% | 11.1% | 11.0% | 11.3% |
| Maximal Educational Attainment | 4.8% | 4.8% | 5.1% | 5.2% |
| Smoker Status | 0.1% | 0.9% | 8.0% | 0.9% |
| History of Diabetes | 0.2% | 3.4% | 11.5% | 13.5% |
| History of CVD | 2.8% | 5.5% | 18.6% | 20.7% |

**Supplementary Figure S3 – Verbal memory and Processing speed scores (Mean +/- 1 standard deviation) for each level of self-reported PA across ages 43y, 53y, 63y and 69y in National Survey of Health and Development (NSHD) participants.**

**
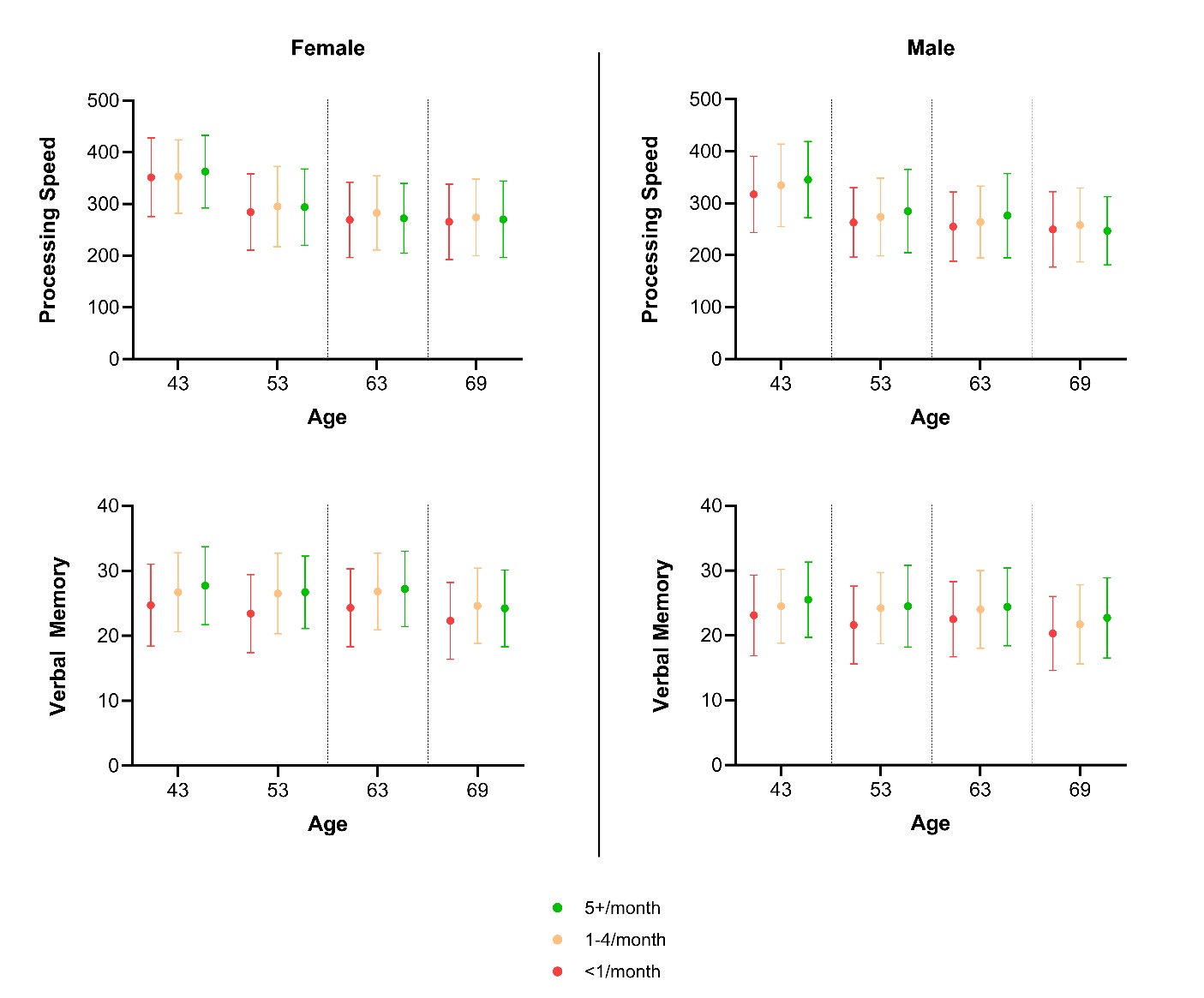
**


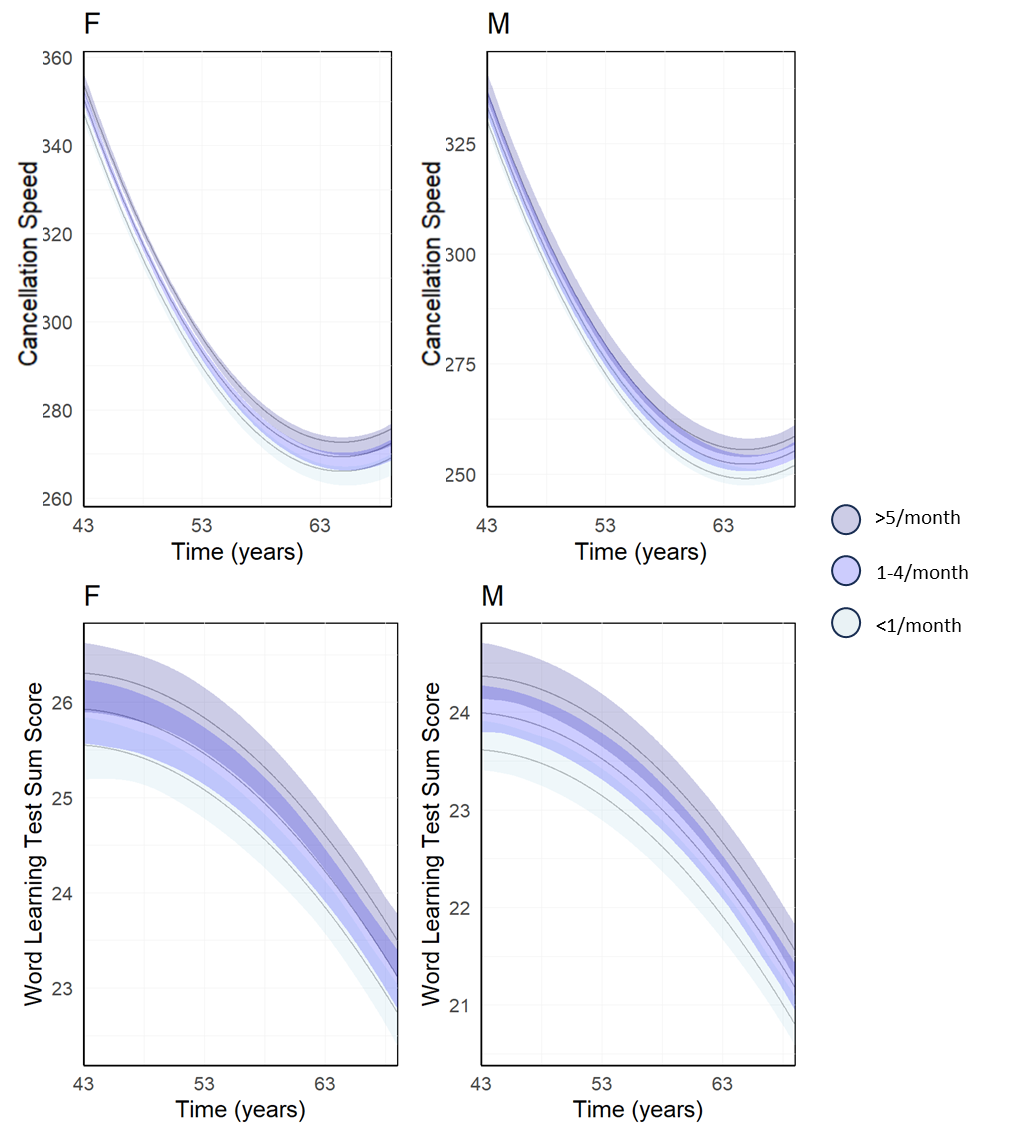
**Supplementary Figure S4 – Change in cognitive scores over time in respect to PA engagement using unadjusted random effects model estimates and bootstrapped 95% CI’s.**

| **Supplementary Table S3. Bivariate CLPM of PA and Verbal memory (VM) - Standardised Path Coefficients** | | | | |
| --- | --- | --- | --- | --- |
| **FEMALE** |  |  |  |  |
| **Autoregressive Paths** | **Standardised Coef.** | **Lower Interval** | **Upper Interval** | **p value** |
| PA 43y **→** PA 53y | 0.32 | 0.26 | 0.38 | **<0.01** |
| PA 53y **→** PA 63y | 0.35 | 0.27 | 0.43 | **<0.01** |
| PA 63y **→** PA 69y | 0.53 | 0.45 | 0.61 | **<0.01** |
| PA 43y **→** PA 63y | 0.21 | 0.13 | 0.29 | **<0.01** |
| PA 53y **→** PA 69y | 0.11 | 0.01 | 0.21 | **<0.01** |
| PA 43y **→** PA 69y | 0.05 | -0.01 | 0.11 | 0.17 |
| VM 43y **→** VM 53y | 0.64 | 0.60 | 0.68 | **<0.01** |
| VM 53y **→** VM 63y | 0.42 | 0.36 | 0.48 | **<0.01** |
| VM 63y **→** VM 69y | 0.33 | 0.27 | 0.39 | **<0.01** |
| VM 43y **→** VM 63y | 0.39 | 0.33 | 0.45 | **<0.01** |
| VM 53y **→** VM 69y | 0.31 | 0.25 | 0.37 | **<0.01** |
| VM 43y **→** VM 69y | 0.19 | 0.13 | 0.25 | **<0.01** |
| **Cross-lagged Paths** |  |  |  |  |
| PA 43y **→** VM 53y | 0.05 | 0.01 | 0.09 | **0.04** |
| PA 53y **→** VM 63y | -0.01 | -0.07 | 0.05 | 0.79 |
| PA 63y **→** VM 69y | -0.01 | -0.09 | 0.07 | 0.87 |
| VM 43y **→** PA 53y | 0.21 | 0.15 | 0.27 | **<0.01** |
| VM 53y **→** PA 63y | 0.12 | 0.04 | 0.20 | **<0.01** |
| VM 63y **→** PA 69y | 0.12 | 0.06 | 0.18 | **<0.01** |
| **Covariances** |  |  |  |  |
| PA 43y ⇔ VM 43y | 0.23 | 0.17 | 0.29 | **<0.01** |
| PA 53y ⇔ VM 53y | 0.18 | 0.10 | 0.26 | **<0.01** |
| PA 63y ⇔ VM 63y | 0.13 | 0.03 | 0.23 | **<0.01** |
| PA 69y ⇔ VM 69y | -0.05 | -0.15 | 0.05 | 0.26 |
| **MALE** |  |  |  |  |
| **Autoregressive Paths** | **Standardised Coef.** | **Lower Interval** | **Upper Interval** | **p-value** |
| PA 43y **→** PA 53y | 0.40 | 0.30 | 0.50 | **<0.01** |
| PA 53y **→** PA 63y | 0.39 | 0.29 | 0.49 | **<0.01** |
| PA 63y **→** PA 69y | 0.43 | 0.33 | 0.53 | **<0.01** |
| PA 43y **→** PA 63y | 0.20 | 0.12 | 0.28 | **<0.01** |
| PA 53y **→** PA 69y | 0.15 | 0.05 | 0.25 | **0.01** |
| PA 43y **→** PA 69y | 0.15 | 0.07 | 0.23 | **<0.01** |
| VM 43y **→** VM 53y | 0.62 | 0.58 | 0.66 | **<0.01** |
| VM 53y **→** VM 63y | 0.37 | 0.31 | 0.43 | **<0.01** |
| VM 63y **→** VM 69y | 0.30 | 0.24 | 0.36 | **<0.01** |
| VM 43y **→** VM 63y | 0.43 | 0.37 | 0.49 | **<0.01** |
| VM 53y **→** VM 69y | 0.33 | 0.27 | 0.39 | **<0.01** |
| VM 43y **→** VM 69y | 0.20 | 0.14 | 0.26 | **<0.01** |
| **Cross-lagged Paths** |  |  |  |  |
| PA 43y **→** VM 53y | 0.11 | 0.07 | 0.15 | **<0.01** |
| PA 53y **→** VM 63y | 0.01 | -0.05 | 0.07 | 0.85 |
| PA 63y **→** VM 69y | 0.07 | 0.01 | 0.13 | **0.04** |
| VM 43y **→** PA 53y | 0.1 | 0.04 | 0.16 | **<0.01** |
| VM 53y **→** PA 63y | 0.06 | -0.02 | 0.14 | 0.16 |
| VM 63y **→** PA 69y | 0.1 | 0.02 | 0.18 | **<0.01** |
| **Covariances** |  |  |  |  |
| PA 43y ⇔ VM 43y | 0.17 | 0.11 | 0.23 | **<0.01** |
| PA 53y ⇔ VM 53y | 0.13 | 0.07 | 0.19 | **<0.01** |
| PA 63y ⇔ VM 63y | 0.09 | -0.01 | 0.19 | **0.05** |
| PA 69y ⇔ VM 69y | 0 | -0.10 | 0.10 | 0.97 |
| Associated standardised thresholds of PA*latent Factor for transition between categories on observed categorical PA variable are presented in Supplementary Table S5. | | | | |

**Supplementary Figure S5 – Final, constrained Autoregressive cross-lagged panel models (Processing Speed)**


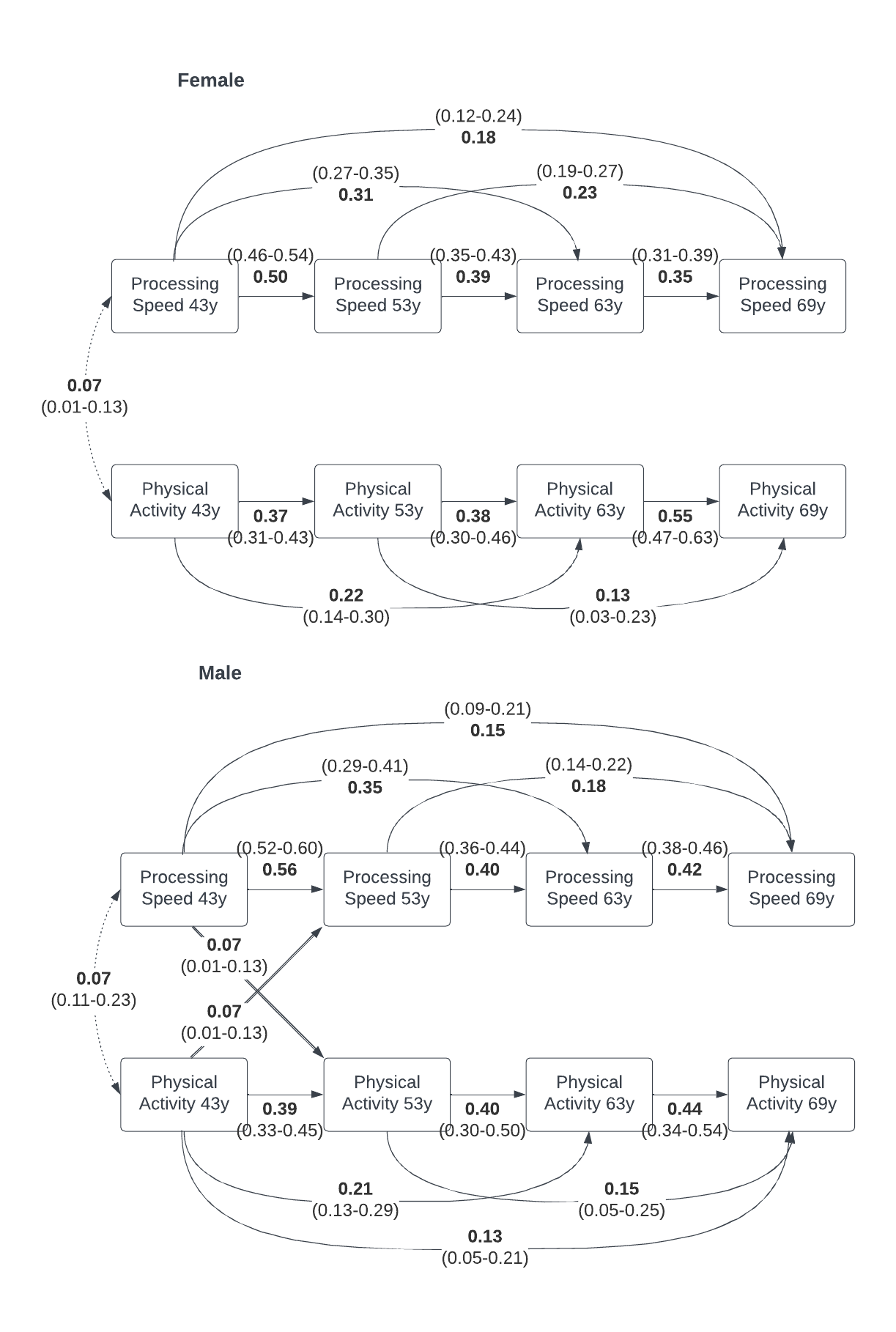


| **Supplementary Table S4. Bivariate CLPM of PA and Processing Speed (PS) - Standardised Path Coefficients** | | | | |
| --- | --- | --- | --- | --- |
| **FEMALE** | **Standardised Coef.** | **Lower CI** | **Upper CI** | **p value** |
| **Autoregressive Paths** |  |  |  |  |
| PA 43y **→** PA 53y | 0.37 | 0.31 | 0.43 | **<0.01** |
| PA 53y **→** PA 63y | 0.38 | 0.30 | 0.46 | **<0.01** |
| PA 63y **→** PA 69y | 0.55 | 0.47 | 0.63 | **<0.01** |
| PA 43y **→** PA 63y | 0.22 | 0.14 | 0.30 | **<0.01** |
| PA 53y **→** PA 69y | 0.13 | 0.03 | 0.23 | **0.01** |
| PA 43y **→** PA 69y | 0.06 | -0.02 | 0.14 | 0.1 |
| PS 43y **→** PS 53y | 0.5 | 0.46 | 0.54 | **<0.01** |
| PS 53y **→** PS 63y | 0.39 | 0.35 | 0.43 | **<0.01** |
| PS 63y **→** PS 69y | 0.35 | 0.31 | 0.39 | **<0.01** |
| PS 43y **→** PS 63y | 0.31 | 0.27 | 0.35 | **<0.01** |
| PS 53y **→** PS 69y | 0.23 | 0.19 | 0.27 | **<0.01** |
| PS 43y **→** PS 69y | 0.18 | 0.12 | 0.24 | **<0.01** |
| **Cross-lagged Paths** |  |  |  |  |
| PA 43y **→** PS 53y | 0 | -0.04 | 0.04 | 0.89 |
| PA 53y **→** PS 63y | 0.02 | -0.04 | 0.08 | 0.59 |
| PA 63y **→** PS 69y | 0.01 | -0.05 | 0.07 | 0.88 |
| PS 43y **→** PA 53y | 0.03 | -0.03 | 0.09 | 0.42 |
| PS 53y **→** PA 63y | -0.02 | -0.10 | 0.06 | 0.64 |
| PS 63y **→** PA 69y | 0.05 | -0.01 | 0.11 | 0.13 |
| **Covariances** |  |  |  |  |
| PA 43y ⇔ PS 43y | 0.07 | 0.01 | 0.13 | **0.01** |
| PA 53y ⇔ PS 53y | 0.04 | -0.02 | 0.10 | 0.21 |
| PA 63y ⇔ PS 63y | 0 | -0.08 | 0.08 | 0.93 |
| PA 69y ⇔ PS 69y | -0.01 | -0.11 | 0.09 | 0.76 |
| **MALE** | **Standardised Coef.** | **Upper CI** | **Lower CI** | **p-value** |
| **Autoregressive Paths** |  |  |  |  |
| PA 43y **→** PA 53y | 0.39 | 0.33 | 0.45 | **<0.01** |
| PA 53y **→** PA 63y | 0.4 | 0.30 | 0.50 | **<0.01** |
| PA 63y **→** PA 69y | 0.44 | 0.34 | 0.54 | **<0.01** |
| PA 43y **→** PA 63y | 0.21 | 0.13 | 0.29 | **<0.01** |
| PA 53y **→** PA 69y | 0.15 | 0.05 | 0.25 | **<0.01** |
| PA 43y **→** PA 69y | 0.13 | 0.05 | 0.21 | **<0.01** |
| PS 43y **→** PS 53y | 0.56 | 0.52 | 0.60 | **<0.01** |
| PS 53y **→** PS 63y | 0.4 | 0.36 | 0.44 | **<0.01** |
| PS 63y **→** PS 69y | 0.42 | 0.38 | 0.46 | **<0.01** |
| PS 43y **→** PS 63y | 0.35 | 0.29 | 0.41 | **<0.01** |
| PS 53y **→** PS 69y | 0.18 | 0.14 | 0.22 | **<0.01** |
| PS 43y **→** PS 69y | 0.15 | 0.09 | 0.21 | **<0.01** |
| **Cross-lagged Paths** |  |  |  |  |
| PA 43y **→** PS 53y | 0.07 | 0.01 | 0.13 | **<0.01** |
| PA 53y **→** PS 63y | 0.03 | -0.03 | 0.09 | 0.34 |
| PA 63y **→** PS 69y | -0.01 | -0.07 | 0.05 | 0.99 |
| PS 43y **→** PA 53y | 0.07 | 0.01 | 0.13 | **0.04** |
| PS 53y **→** PA 63y | 0.06 | -0.02 | 0.14 | 0.14 |
| PS 63y **→** PA 69y | 0.03 | -0.05 | 0.11 | 0.49 |
| **Covariances** |  |  |  |  |
| PA 43y ⇔ PS 43y | 0.17 | 0.11 | 0.23 | **<0.01** |
| PA 53y ⇔ PS 53y | 0.05 | -0.01 | 0.11 | 0.1 |
| PA 63y ⇔ PS 63y | 0.07 | -0.03 | 0.17 | 0.14 |
| PA 69y ⇔ PS 69y | 0.08 | -0.02 | 0.18 | 0.11 |
| Associated standardised thresholds of PA*latent Factor for transition between categories on observed categorical PA variable are presented in Supplementary Table S5. | | | | |

| **Supplementary Table S5. Standardised Thresholds of PA*latent Factor for transition between categories on observed categorical PA variable.** | | | | |
| --- | --- | --- | --- | --- |
| **Transition point on y* latent PA** | **Male** | | **Female** | |
|  | **Threshold (VERBAL MEMORY model)** | **Threshold (Speed model)** | **Threshold (VERBAL MEMORY model)** | **Threshold (Speed model)** |
| PA53 0-1 | 0.150 | 0.257 | 0.189 | 0.304 |
| PA53 1-2 | 0.653 | 0.759 | 0.608 | 0.724 |
| PA63 0-1 | 0.561 | 0.709 | 0.502 | 0.62 |
| PA63 1-2 | 0.957 | 1.107 | 0.917 | 1.038 |
| `PA69 0-1 | 0.441 | 0.569 | 0.438 | 0.526 |
| PA69 1-2 | 0.753 | 0.882 | 0.823 | 0.913 |

| **Supplementary Table S6. Associations between physical activity scores and lagged (prior) cognition.** | | | | | | | | | | | | |
| --- | --- | --- | --- | --- | --- | --- | --- | --- | --- | --- | --- | --- |
|  |  |  | **DV: Physical Activity *(Reference: Less than Monthly)*** | | | | | | | | | |
|  |  |  | **1-4/Month** | | | |  | **5+/Month** | | | |  |
| **Adjustments** | **IV** |  | **RRR** | ***95% CI*** | | ***p-value*** |  | **RRR** | ***95% CI*** | | ***p-value*** |  |
|  | Processing Speed - Z-score (Female N=1467) |  | 1.74 | 0.90 | 1.21 | 0.837 |  | 1.21 | 0.99 | 1.41 | 0.339 |  |
|  |  |  |  |  |  |  |  |  |  |  |  |  |
| **Model 1**  Adjusted for time (centred at 53) | Processing Speed – Z-score (Male N=1421) |  | 1.33 | 1.16 | 1.54 | **<0.01** |  | 1.76 | 1.51 | 2.04 | **<0.01** |  |
|  |  |  |  |  |  |  |  |  |  |  |  |  |
|  | Word Learning Z-score (Female N=1,467) |  | 1.63 | 1.47 | 1.82 | **<0.01** |  | 1.86 | 1.66 | 2.09 | **<0.01** |  |
|  |  |  |  |  |  |  |  |  |  |  |  |  |
|  | Word Learning Z-score (Male N=1,421) |  | 1.48 | 1.32 | 1.67 | **<0.01** |  | 1.63 | 1.44 | 1.84 | **<0.01** |  |
|  |  |  |  |  |  |  |  |  |  |  |  |  |
|  | Processing Speed - Z-score (Female N=1467) |  | 0.96 | 0.83 | 1.11 | 0.257 |  | 1.11 | 0.95 | 1.28 | 0.474 |  |
|  |  |  |  |  |  |  |  |  |  |  |  |  |
| **Model 2**  Further adjusted for childhood cognition, socio-economic position and education. | Processing Speed - Z-score (Male N=1421) |  | 1.22 | 1.06 | 1.42 | **0.007** |  | 1.57 | 1.36 | 1.83 | **<0.01** |  |
|  |  |  |  |  |  |  |  |  |  |  |  |  |
|  | Word Learning Z-score (Female N=1,467) |  | 1.32 | 1.17 | 1.47 | **<0.01** |  | 1.51 | 1.33 | 1.75 | **<0.01** |  |
|  |  |  |  |  |  |  |  |  |  |  |  |  |
|  | Word Learning Z-score (Male N=1,421) |  | 1.28 | 1.12 | 1.45 | **<0.01** |  | 1.34 | 1.18 | 1.54 | **<0.01** |  |
|  |  |  |  |  |  |  |  |  |  |  |  |  |
|  | Processing Speed - Z-score units (Female N=1467) |  | 0.95 | 0.82 | 1.01 | 0.412 |  | 1.08 | 0.93 | 1.25 | 0.598 |  |
|  |  |  |  |  |  |  |  |  |  |  |  |  |
| **Model 3**  Further adjusted for adult history of CVD, diabetes and smoking status, attrition and mortality. | Processing Speed – Z-score (Male N=1421) |  | 1.19 | 1.02 | 1.37 | **0.023** |  | 1.50 | 1.30 | 1.74 | **<0.01** |  |
|  |  |  |  |  |  |  |  |  |  |  |  |  |
|  | Word Learning Z-score (Female N=1,467) |  | 1.30 | 1.15 | 1.46 | **<0.01** |  | 1.48 | 1.31 | 1.68 | **<0.01** |  |
|  |  |  |  |  |  |  |  |  |  |  |  |  |
|  | Word Learning Z-score (Male N=1,421) |  | 1.24 | 1.10 | 1.41 | **<0.01** |  | 1.30 | 1.15 | 1.48 | **<0.01** |  |
|  |  |  |  |  |  |  |  |  |  |  |  |  |

| **Supplementary Table S7. Associations between lagged (prior) physical activity and cognitive domain scores.** | | | | | | | | | | | | | | | | | | | | | | | | |  | |
| --- | --- | --- | --- | --- | --- | --- | --- | --- | --- | --- | --- | --- | --- | --- | --- | --- | --- | --- | --- | --- | --- | --- | --- | --- | --- | --- |
|  |  |  | **DV: Processing Speed-10 score (F) n=1,444** | | | |  | **DV: Processing Speed-10 score (M) n=1,381** | | | |  | **DV: Word Learning Test score (F) n=1,447** | | | | |  | | **DV: Word Learning Test score (M) n=1,385** | | | | | | |
|  |  |  |  |  |  |  |  |  |  |  |  |  |  |  |  |  |  |  | |  |  |  |  |  |  |  |
| **Adjustments** | **IV** |  | ***B*** | ***95% CI*** | | ***p-value*** |  | ***B*** | ***95% CI*** | | ***p-value*** |  | ***B*** | ***95% CI*** | | ***p-value*** |  | | ***B*** | | ***95% CI*** | | ***p-value*** |  | |  |
| **Model 1** (Adjusted for time) | PA <Monthly (Reference) |  | - | - | - | - |  | - | - | - | - |  | - | - |  | - |  | | - | | - | - | - |  | |  |
|  |  |  |  |  |  |  |  |  |  |  |  |  |  |  |  |  |  | |  | |  |  |  |  | |  |
|  | PA 1-4/Month |  | -0.01 | -0.08 | 0.05 | 0.71 |  | 0.13 | 0.06 | 0.20 | **<0.01** |  | 0.13 | 0.06 | 0.19 | **<0.01** |  | | 0.14 | | 0.07 | 0.21 | **<0.01** |  | |  |
|  |  |  |  |  |  |  |  |  |  |  |  |  |  |  |  |  |  | |  | |  |  |  |  | |  |
|  | PA 5+/Month |  | 0.15 | -0.05 | 0.17 | 0.66 |  | 0.15 | 0.08 | 0.22 | **<0.01** |  | 0.10 | 0.04 | 0.17 | **<0.01** |  | | 0.20 | | 0.13 | 0.26 | **<0.01** |  | |  |
|  |  |  |  |  |  |  |  |  |  |  |  |  |  |  |  |  |  | |  | |  |  |  |  | |  |
| **Model 2** (Further adjusted for education and childhood factors) | PA <Monthly (Reference) |  | - | - | - | - |  | - | - | - | - |  | - | - |  | - |  | | - | | - | - | - |  | |  |
|  |  |  |  |  |  |  |  |  |  |  |  |  |  |  |  |  |  | |  | |  |  |  |  | |  |
|  | PA 1-4/Month |  | -0.04 | -0.10 | 0.03 | 0.30 |  | 0.11 | 0.03 | 0.17 | **<0.01** |  | 0.08 | 0.02 | 0.14 | **0.02** |  | | 0.09 | | 0.02 | 0.16 | **<0.01** |  | |  |
|  |  |  |  |  |  |  |  |  |  |  |  |  |  |  |  |  |  | |  | |  |  |  |  | |  |
|  | PA 5+/Month |  | -0.01 | -0.08 | 0.05 | 0.73 |  | 0.12 | 0.05 | 0.19 | **<0.01** |  | 0.04 | -0.01 | 0.10 | 0.17 |  | | 0.14 | | 0.08 | 0.21 | **<0.01** |  | |  |
|  |  |  |  |  |  |  |  |  |  |  |  |  |  |  |  |  |  | |  | |  |  |  |  | |  |
| **Model 3** (Further adjusted for adult health factors, attrition and mortality). | PA <Monthly (Reference) |  | - | - | - | - |  | - | - | - | - |  | - | - |  | - |  | | - | | - | - | - |  | |  |
|  |  |  |  |  |  |  |  |  |  |  |  |  |  |  |  |  |  | |  | |  |  |  |  | |  |
|  | PA 1-4/Month |  | -0.04 | -0.10 | 0.03 | 0.29 |  | 0.09 | 0.02 | 0.16 | **0.01** |  | 0.08 | 0.01 | 0.14 | **0.02** |  | | 0.08 | | 0.01 | 0.15 | **0.02** |  | |  |
|  |  |  |  |  |  |  |  |  |  |  |  |  |  |  |  |  |  | |  | |  |  |  |  | |  |
|  | PA 5+/Month |  | -0.01 | -0.07 | 0.05 | 0.69 |  | 0.10 | 0.03 | 0.16 | **<0.01** |  | 0.04 | -0.02 | 0.10 | 0.23 |  | | 0.13 | | 0.06 | 0.19 | **<0.01** |  | |  |
|  |  |  |  |  |  |  |  |  |  |  |  |  |  |  |  |  |  | |  | |  |  |  |  | |  |

| **Supplementary Table S8. Interaction between lagged (prior) cognition and an age-69 indicator variable in random effects models.** | | | | | |
| --- | --- | --- | --- | --- | --- |
| **Interaction** |  | **RRR** | **95% C.I.** | | **p-value** |
| **Female - DV: PA** | | | | | |
| Model 1: (Unadjusted)  Lagged VERBAL MEMORY*Age 69 | PA: 1-4/month | 1.00 | 0.96 | 1.04 | 0.86 |
|  | PA: 5+/month | 1.03 | 0.99 | 1.06 | 0.18 |
| **Male - DV: PA** | | | | | |
| Model 1: (Unadjusted)  Lagged Speed*Age 69 | PA: 1-4/month | 1.01 | 0.97 | 1.05 | 0.60 |
|  | PA: 5+/month | 1.00 | 0.97 | 1.03 | 0.91 |
| Lagged VERBAL MEMORY*Age 69 | PA: 1-4/month | 1.06 | 0.94 | 1.03 | 0.54 |
|  | PA: 5+/month | 1.04 | 1.00 | 1.08 | **0.04** |
| Model 2: (Further adjusted for education and childhood factors)  Lagged VERBAL MEMORY*Age 69 | PA: 1-4/month | 0.99 | 0.95 | 1.03 | 0.52 |
|  | PA: 5+/month | 1.04 | 1.00 | 1.08 | **0.036** |
| Model 3: (Fully-adjusted)  Lagged VERBAL MEMORY*Age 69 | PA: 1-4/month | 0.99 | 0.94 | 1.03 | 0.532 |
|  | PA: 5+/month | 1.04 | 1.00 | 1.08 | **0.046** |
| VERBAL MEMORY: Word Learning Task; PA: Physical Activity; RRR: Relative Risk Ratio; C.I.: Confidence Interval. WLT is in 1-word units, Cancellation speed is in 10-letter units. | | | | | |

| **Supplementary Table S9. Interaction between lagged (prior) PA and an age-69 indicator variable in random effects models.** | | | | |
| --- | --- | --- | --- | --- |
| **Female - DV: Processing Speed (10-letter units)** | | | | |
| Lagged PA(1)*Age 69 | -0.47 | -1.66 | 0.72 | 0.44 |
| Lagged PA(2)*Age 69 | 0.21 | -0.79 | 1.21 | 0.69 |
| **Female - DV: VERBAL MEMORY (1-word units)** | | | | |
| Lagged PA*Age 69 | -0.10 | -0.98 | 0.78 | 0.83 |
| Lagged PA*Age 69 | -0.39 | -1.12 | 0.35 | 0.30 |
| **Male - DV: Processing Speed (10-letter units)** | | | | |
| Lagged PA*Age 69 | -0.64 | -1.88 | 0.61 | 0.32 |
| Lagged PA*Age 69 | -6.97 | -1.72 | 0.33 | 0.18 |
| **Male - DV: VERBAL MEMORY (1-word units)** | | | | |
| Lagged PA*Age 69 | 0.06 | -0.87 | 1.00 | 0.89 |
| Lagged PA*Age 69 | 0.60 | -0.17 | 1.36 | 0.13 |
| VERBAL MEMORY: Word Learning Task; PA: Physical Activity; RRR: Relative Risk Ratio; C.I.: Confidence Interval. | | | | |

| **Supplementary Table S10. Participant Characteristics at Baseline (age 43)** | | | | | | |
| --- | --- | --- | --- | --- | --- | --- |
|  |  | **Included** | | **Excluded** | |  |
| **Total** | N | **2888** | | **2474** | | **p-value** |
| **Sociodemographic Factors** |  |  |  |  |  |  |
| **Sex (Female)** | N (%) | 1467 | 50.5 | 1079 | 43.6 | **<0.01** |
| **Parental Social Class** |  |  |  |  |  | **<0.01** |
| IV Partly Skilled/V-Unskilled manual | | 748 | 24.3 | 526 | 27.2 |  |
| III-Skilled non-manual or manual | | 1324 | 48.4 | 959 | 49.6 |  |
| I-Professional/II-Intermediate |  | 666 | 27.3 | 448 | 23.2 |  |
| **Childhood Confounders** | |  |  |  |  |  |
| **Age 5 Cognition (Standardised)** | Mean (SD) | 0.08 | 0.8 | -0.12 | 0.9 | **<0.01** |
| **Maximal Educational Attainment** | N (%) |  |  |  |  | **<0.01** |
| None/Below O-level/GCSE (Less than age 16) | | 947 | 34.6 | 818 | 48.4 |  |
| O-level/GCSE or Vocational Qualification | 783 | 28.5 | 432 | 25.5 | 17.5 |  |
| A-level or equivalent (Up to age 18)/Degree or higher degree | | 1009 | 36.8 | 442 | 26.1 |  |
| **Health & Lifestyle Factors** | |  |  |  |  |  |
| **Smoker Status** | N (%) |  |  |  |  | **<0.01** |
| Never |  | 831 | 29.9 | 117 | 24.7 |  |
| Ex Smoker |  | 1179 | 42.4 | 151 | 31.9 |  |
| Current Smoker |  | 767 | 27.7 | 206 | 43.4 |  |
| **History of Diabetes** | N (%) | 24 | 0.9 | 11 | 2.0 | **<0.01** |
| **History of CVD** | N (%) | 87 | 3.2 | 15 | 3.2 | 0.9 |
| Reported percentages are prior to imputation and therefore totals for each covariate vary depending on variable missingness. (Categorical variables examined by Chi-squared tests; continuous normally distributed by t-test) | | | | | | |

1. James S-N, Chiou Y-J, Fatih N, Needham LP, Schott JM, Richards M. Timing of physical activity across adulthood on later-life cognition: 30 years follow-up in the 1946 British birth cohort. Journal of Neurology, Neurosurgery &amp; Psychiatry. 2023;94(5):349-56.

2. Galobardes B, Lynch J, Smith GD. Measuring socioeconomic position in health research. Br Med Bull. 2007;81-82:21-37.

3. Walsh JJ, Barnes JD, Cameron JD, Goldfield GS, Chaput JP, Gunnell KE, et al. Associations between 24 hour movement behaviours and global cognition in US children: a cross-sectional observational study. Lancet Child Adolesc Health. 2018;2(11):783-91.

4. Muthén B, Asparouhov T, Witkiewitz K. Cross-Lagged Panel Modeling with Categorical Outcomes. 2023.

5. Li C-H. Statistical estimation of structural equation models with a mixture of continuous and categorical observed variables. Behavior Research Methods. 2021;53(5):2191-213.

6. Rhemtulla M, Brosseau-Liard P, Savalei V. When can categorical variables be treated as continuous? A comparison of robust continuous and categorical SEM estimation methods under suboptimal conditions. Psychol Methods. 2012;17(3):354-73.

7. Selig J, Little T. Autoregressive and cross-lagged panel analysis for longitudinal data. 2012. p. 265-78.
